# Doped Ferrite Nanoparticles Exhibiting Self-Regulating Temperature as Magnetic Fluid Hyperthermia Antitumoral Agents, with Diagnostic Capability in Magnetic Resonance Imaging and Magnetic Particle Imaging

**DOI:** 10.3390/cancers14205150

**Published:** 2022-10-20

**Authors:** Federica Vurro, Marco Gerosa, Alice Busato, Matilde Muccilli, Emil Milan, Jeff Gaudet, Patrick Goodwill, James Mansfield, Enrico Forlin, Alessandro Negri, Filippo Gherlinzoni, Giovanni Morana, Michele Gottardi, Paolo Matteazzi, Max Wintermark, Adolfo Speghini, Pasquina Marzola

**Affiliations:** 1 Department of Computer Science, University of Verona, Strada Le Grazie 15, 37134 Verona, Italy; 2 Department of Diagnostics and Public Health, University of Verona, Piazzale L.A. Scuro, 37134 Verona, Italy; 3 Nanomaterials Research Group, Department of Biotechnology, University of Verona and INSTM, RU Verona, Strada Le Grazie 15, 37134 Verona, Italy; 4 Magnetic Insight Inc., Alameda, CA 94501, USA; 5 M.B.N. Nanomaterialia S.p.A., Via Giacomo Bortolan, 42, 31050 Carbonera Treviso, Italy; 6 Foundation for Nanotheranostics Research in Cancer Therapy, RNC, 31100 Treviso, Italy; 7 Department of Radiology, Stanford University Hospital, Stanford, CA 94305, USA

**Keywords:** magnetic fluid hyperthermia, self-regulating temperature, MRI, MPI, breast cancer

## Abstract

**Simple Summary:**

Hyperthermia, a limited increase in tumor tissue temperature up to a maximum of 45 °C, has long been used in cancer treatment, either as a stand-alone therapy or in conjunction with chemo-/radiotherapy. Among the different physical procedures used, a particularly promising technique is magnetic fluid hyperthermia (MFH): this consists of the local administration of magnetic nanoparticles, followed by the application of alternating magnetic fields. One concern with this technique is the possibility of damage to healthy peritumoral tissue. The present study investigates innovative nanoparticles with self-regulating temperature, which should reduce this risk and thus mark a significant step forward in MFH. In an experimental model of aggressive breast cancer, we demonstrated a substantial reduction of tumor growth rate by using an experimental MFH protocol, transferable to clinical practice. These innovative nanomaterials present the added advantage of allowing non-invasive monitoring of temperature, by magnetic resonance imaging (MRI) and magnetic particle imaging (MPI).

**Abstract:**

This paper reports a comprehensive investigation of a magnetic nanoparticle (MNP), named M55, which belongs to a class of innovative doped ferrite nanomaterials, characterized by a self-limiting temperature. M55 is obtained from M48, an MNP previously described by our group, by implementing an additional purification step in the synthesis. M55, after citrate and glucose coating, is named G-M55. The present study aimed to demonstrate the properties of G-M55 as a diagnostic contrast agent for MRI and magnetic particle imaging (MPI), and as an antitumoral agent in magnetic fluid hyperthermia (MFH). Similar specific absorption rate values were obtained by standard MFH and by an MPI apparatus. This result is of interest in relation to the application of localized MFH by MPI apparatus. We demonstrated the biocompatibility of G-M55 in a triple-negative human breast cancer line (MDA-MB-231), and its efficacy as an MFH agent in the same cell line. We also demonstrated the efficacy of MFH treatment with G-M55 in an experimental model of breast cancer. Overall, our results pave the way for the clinical application of G-M55 as an MFH agent in breast cancer therapy, allowing not only efficient treatment by both standard MFH apparatus and MPI but also temperature monitoring.

## 1. Introduction

Breast cancer is one of the leading causes of cancer morbidity and mortality. A recent review reported that it is the second most diagnosed malignancy, accounting for more than 11.6% of female cancers [1,2], and the fifth most common cause of cancer deaths, making up 6.6% of all cancer mortality worldwide. The same review reports that breast cancer induces a substantial public health burden, leading to a loss of 14.8 million Disability Adjusted Life Years (DALYs). Despite progress in early diagnosis of the disease and the variety of therapeutic approaches available, it is still the most frequently reported cause of female cancer deaths.

Nowadays, primary mammary tumors can be treated by surgery, followed by chemotherapy or radiotherapy—an invasive and debilitating approach, contributing to the above-mentioned public health burden. Chemotherapy and radiotherapy can damage healthy tissues, while surgery entails risks of infection and bleeding and is not always curative for metastatic tumors. Innovative, minimally invasive treatment methods, devoid of negative impacts, are desirable to reduce mortality and morbidity in breast cancer patients. One candidate is hyperthermia, which can be generated from sources such as ultrasound, RF waves and laser beams [3,4]. Recent advances in nanotechnology have paved the way for magnetic fluid hyperthermia (MFH), combining magnetic nanoparticles (MNPs) with alternating magnetic fields (AMFs). MFH has shown great promise in cancer research, with the potential to overcome many of the limitations presented by other hyperthermia techniques [5].

The challenges of MFH are the need to deliver a large quantity of MNPs to the tumor tissue, and the risk of overheating healthy peritumoral tissue [6]. In currently applied preclinical and clinical protocols, nanoparticles are injected directly into the tumor tissue. For tumors in anatomical regions not readily accessible by injection, an invasive procedure entailing surgery and anesthesia may be required. This is not the case for breast tumors: given that image-guided biopsy is routinely performed in breast cancer, the intratumoral injection of MNPs for MFH treatment could be easily carried out as an outpatient procedure, thus avoiding invasive surgery.

For the treatment of tumors located deep in the body, systemic administration of MNPs could be envisaged, although considerably limited by their non-specific uptake to the liver and spleen, typically exceeding specific uptake to the tumor. In this respect, the application of magnetic particle imaging (MPI) to excite MNPs in specific regions of the body has been reported [4,5,7]. The field-free region of MPI instrumentation, which can be localized over the tumor with millimetric precision, allows space-selective irradiation of the tumor region. This makes MFH by MPI apparatus compatible with systemic MNP administration, without risk of damage to the liver and spleen, provided sufficient MNPs are delivered to tumor tissue.

The risk of overheating healthy peritumoral tissue has been previously addressed by us and by other groups [6,8] using self-regulating temperature MNPs [9]. Gerosa et al. report an extensive investigation of a self-regulating temperature nanomaterial, synthesized by MBN Nanomaterialia S.p.A. (Treviso, Italy). Such nanomaterials undergo a stable temperature increase when exposed to an AMF, up to a maximum value (TSR, self-regulating temperature) lower than the Curie temperature (T_C_). T_C_ values can be modulated by adjusting the composition of the nanomaterial. Above the T_C_, magnetization is negligible and the heating effect of the AMF ceases. Interestingly, above Tc, a sharp decrease in transverse MRI relaxivity occurs, meaning that the MNP is suitable for use as a temperature-sensitive contrast agent [6]. In principle, these properties are particularly useful when the nanomaterial is integrated into biological matrices, making it possible to limit the maximum temperature and, at the same time, monitor temperature changes by means of imaging.

M55, the MNP studied in the present paper, is obtained from previously described M48 material [6], by implementing an additional purification step in the synthesis process so as to remove potential paramagnetic by-products from the liquid suspension. In investigating this class of materials, we have already considered their properties as theranostic agents for MPI, in view of the foreseen application of MPI in MFH [4,7]. The capability of M55 as a bimodal contrast agent was demonstrated in vitro, and in a cellular imaging experiment. In addition, we demonstrated the marked therapeutic efficacy of M55 in an experimental protocol, in which four MFH treatments are applied after a single intratumoral injection of MNPs.

## 2. Materials and Methods

### 2.1. Synthesis of the Iron-Based NPs

The NPs were prepared by MBN Nanomaterialia S.p.A. (Treviso, Italy) following a proprietary mechanochemical process [9]. Briefly, MgO, Fe_2_O_3,_ and TiO_2_ powders with a 4:2:1 molar ratio, respectively, were thoroughly mixed, and the reaction was carried out using ball milling equipment [10] for 6 h. A heat treatment at 1200 °C for 4 h in the air was then carried out. The obtained powders were finely ball-milled for 1 h, then homogeneously dispersed in isopropanol and ultrasonicated to promote de-aggregation. Centrifugation was used to extract nanosized particles (<200 nm). This process further improves the one previously used [6]: detailed processing parameters are provided courtesy of MBN Nanomaterialia S.p.A.

### 2.2. Structural Characterization

The phase and crystal characterization were analyzed by X-ray powder diffraction (XRPD) technique. The X-ray diffraction pattern was measured using an X-ray powder diffractometer (Thermo ARLX’TRA, Waltham, MA, USA) in Bragg–Brentano geometry (Cu source, Si (Li)-cooled solid-state detector) with a scan rate of 0.09°/min. The sample was homogenized in an agate mortar adding some drops of ethanol. The sample was then deposited on a low background stage. The ICSD database was used for phase analysis.

### 2.3. Morphological Analysis and Dimensional Characterization

The morphology of the NPs was analyzed with transmission electron microscopy (TEM, FEI TECNAI G2). The sample was deposited on a copper grid and then desiccated for one day before the measurements.

The hydrodynamic size and zeta potential of the coated nanoparticles were determined with a Malvern Zetasizer Nano instrument. The sample was prepared in an appropriate plastic cuvette, repeating the size and zeta potential measurements for the following 7 days to assess the stability of the coating.

### 2.4. Coating of the Iron-Based NPs

The NPs were coated with citrate ions, using sodium citrate dihydrate (99%—Alfa Aesar, Haverhill, MA, USA) and glucose (D-(+)-glucose SigmaUltra 99.5%) with a procedure described in [6]. The citrate- and glucose-coated NPs will be referred to below as G-M55.

### 2.5. Analysis of the NPs Coating

The NPs coating was analyzed by FT-IR spectroscopy with a JASCO FT/IR-660 plus spectrometer. The sample was prepared by dispersing 3 mg of powders of G-M55 in 300 mg KBr and preparing a pellet of 1 cm diameter.

### 2.6. Cytotoxicity Assay

MDA-MB-231 cells were cultured in complete culture medium Dulbecco’s Modified Eagle’s Medium (DMEM) containing 10% Fetal Bovine Serum (FBS), 1% of a mix of penicillin/streptomycin 1:1 (GIBCO Life Technology, Monza, Italy), and 1% L-glutamine 200 mM (GIBCO Life Technology, Monza, Italy). Then, 3000 cells were seeded in each well of a 96-well plate and incubated for 24 h at 37 °C in humidified air with 5% CO_2_. The medium was then replaced with fresh medium, containing 10, 50, 100, 150, and 300 µg/mL of G-M55.

Cytotoxicity was assessed through the MTT (3-(4,5-dimethylthiazol-2-yl)-2,5-diphenyltetrazolium bromide) assay after 2, 24, and 48 h of incubation. Then, 100 µL of MTT solution (5 mg/mL) was added to each well and incubated for an additional 4 h. Then, the MTT solution was removed and 100 µL of DMSO was added to each well to dissolve the formazan crystal. Absorbance was read using an HTX microplate reader (BioTek Instruments, Winooski, VT, USA) at a wavelength of 570 nm. Four measurements of optical density (OD) were recorded for each sample, and cell viability (%) was calculated with the following equation: CV% = (ODsample/ODcontrol) × 100.

### 2.7. Magnetic Characterization

The efficiency of G-M55 as a multimodal contrast agent was assessed in MPI by using a RELAX module (Magnetic Insight Inc., Alameda, CA, USA), and in MRI by using a 7T Bruker Biospec system. In MPI, two components of the measurement were assessed: the signal efficiency was measured through the amount of MPI signal per mass of iron (high signal efficiency results in improved SNR and lower detection limits) and full width at half-maximum (FWHM), which is related to the imaging resolution (lower FWHM results in less signal spread and improved imaging resolution). The resolution, defined as the distance for 2 objects separated at 50%, was estimated according to the following relationship: Resolution (mm) = FWHM (mT)/Gradient (T/m). A sample containing G-M55 with a concentration of 5.5 μg of Fe in 10.0 μL volume was vortexed for 5 min and sonicated in a room temperature water bath sonicator for 5 min before testing. Relaxometry measurements were performed, using the RELAX module on naked and coated nanoparticles at the same concentration, and compared to VivoTrax (Ferucarbotran, Magnetic Insight Inc., Alameda, CA, USA). The 1D signal acquisition mode was used for relaxometry measurements. A 2D projection image was used to test the nanoparticles with the following imaging parameters: standard mode, coronal acquisition, 2-channel, FOV = 6 × 6 cm^2^.

In order to measure transverse relaxivity (spin-spin relaxation process) in MRI, the transverse relaxation time *T*_2_ of water containing different amounts of G-M55 was measured by acquiring a MSME (multi-slice multi-echo) pulse sequence, with the following parameters: TR = 2000 ms, TE from 6.5 to 170 ms, FOV = 55 × 55 mm, matrix size = 128 × 128, slice thickness = 1 mm, number of echoes = 25. The signals coming from each phantom at different TEs were averaged to obtain the relaxation curve decay, which was fitted with a single exponential decay function to obtain the *T*_2_ value. Transverse relaxivity *r*_2_ was then obtained by measuring the modified relaxation time of water through the presence of G-M55 at different NP concentrations, and extrapolated via linear fit using the following equation:

(1) $$\frac{1}{T_{2}}=\frac{1}{T_{2,0}}{+r}_{2}c$$

where *T*_2,0_ is the relaxation time of pure water, *c* the concentration of G-M55 (expressed as mM of Fe), and *T*_2_ the modified relaxation time of water.

### 2.8. Multimodal Imaging of Cells Labeled with NPs

To test the efficiency of G-M55 as an MPI contrast agent in a biologically significant context, it was used in a cellular imaging experiment. MDA-MB-231 cells were seeded and cultured in DMEM with 10% FBS, 1% penicillin/streptomycin 1:1 mix, and 1% L-glutamine 200 mM, in a T-75 flask (5 × 10^6^ cells) and accommodated for 24 h. MDA-MB-231 cells were incubated with 0.1 mgFe/mL (corresponding to 0.256 mg/mL of MNP) of G-M55 for 24 h, to observe iron uptake. Cells were then washed 3 times with 5.0 mL of phosphate buffered saline solution (PBS) and detached with 1.5 mL of 0.25% trypsin, harvested, and centrifuged (1200 rpm for 3 min). After collecting and throwing out the supernatant, the pellet was resuspended in 1.0 mL of PBS. The sample was divided into three portions of 4 × 10^6^ cells in 0.1 mL PBS each, for MPI. Serial dilutions were performed, to obtain samples containing decreasing amounts of cells (from 4 × 10^6^ to 30 × 10^3^ cells).

Images of the pellets were acquired with a Momentum MPI scanner (Magnetic Insight Inc., Alameda, CA, USA), using a 2D projection mode scan with the default mode setting (5.7 T/m gradient). The imaging parameters adopted were: field of view (FOV) = 4 × 12 cm^2^; single average; acquisition time = 10.0 s for each projection; reconstruction time = 4.0 min.

For quantitative MPI analysis, the cell pellet was outlined in a single slice with a freehand region of interest (ROI). The MPI signal was calculated using the sum function of the VivoQuant software; this function calculates the sum of pixel signal intensity within a given ROI, normalized to the number of pixels. A calibration curve of the tracer was set up by 2D scanning of NPs at different concentrations, expressed as iron content. A duplicate of each dilution was scanned.

After computing the calibration curves, the MPI signal collected from the cell pellets was converted into iron content, using the following equation based on the calibration curves and the doses employed:

(2) $$Fe_{\mu g}=Fe_{incubated}\frac{\frac{Sum_{Tissue}}{Surface_{Tissue}}}{\frac{Sum_{Tracer}}{Surface_{Tracer}}}$$

Cell pellets were also prepared in the bottom of Eppendorf tubes, for MRI. A gradient echo acquisition sequence was used, with the following parameters: TR = 460 ms, TE = 5 ms, flip angle = 30°, FOV = 43 × 43 mm^2^, matrix size = 384 × 384, NEX = 6.

### 2.9. Magnetic Fluid Hyperthermia and Magnetic Particle Imaging HYPER Module

Magnetic nanoparticles, in the presence of an external AMF, convert magnetic energy into heat. This ability is quantified from the value of the specific absorption rate (*SAR*). The *SAR* value of nanoparticles in solution is calculated by using the following equation:

(3) $$SAR=\frac{∆T}{∆t}\frac{C}{m_{Fe}}$$

where *C* is the specific heat capacity of the solvent (4.186 J/g°C), ∆*T* is temperature variation (°C), Δ*t* is the time interval (s), and mFe is the iron mass fraction in the compound. Temperature variation was calculated over the first 60 s of heating. *SAR* was measured using a Nanotherics MagneTherm system (Warrington, UK), with samples diluted in water. The heat dissipation value depends on the frequency and amplitude of the AMF. The AMF apparatus yields a maximum magnetic field intensity of 23.0 kA/m (approximately 29.0 mT). A multichannel thermometer equipped with optical fiber probes (FOTEMP4, Optocon AG, Dresden, Germany) was used to assess temperature variation within the sample, every 10.0 s. The multichannel thermometer was placed inside the sample throughout the entire acquisition time. MNP concentration was 6 mg/mL. The specific time window was chosen considering previous results obtained with G-M48 [6], as well as translatability to clinical practice. The characteristic parameters of AMF were chosen in order to satisfy the Hergt criterion for clinical translatability [11]: in particular, magnetic field intensity was set at H = 8.75 kA/m, with a frequency of 473.1 kHz. The samples were kept in a homemade closed box with a thermostatic air stream at 37 °C.

*SAR* was also measured with the HYPER module of the Magnetic Particle Imager Momentum scanner (Magnetic Insight Inc., Alameda, CA, USA). The change in temperature over time was recorded for both coated and naked nanoparticles, for 300 s (the necessary time window to evaluate *SAR*), starting at thermostated room temperature with a magnetic field excitation of 12.75 kA/m at a 1600 kA/m^2^ gradient. The samples used had a volume of 100 µL, with the same *Fe* concentration as for the measurement with the Nanotherics system.

MFH was also performed in cell culture as previously described [6]. Briefly, cells were incubated with 150 µg/mL of G-M55, and after 24 h of incubation, cells were placed in a homemade closed box with a thermostatic air stream for 15 min and then an external AMF (MFH treatment) was applied to the cells for an additional 20 min. The efficiency of the MFH treatment was assessed through the MTT assay as described in Section 2.6. Cells subjected to MFH and cells incubated with 150 µg/mL of G-M55, but not exposed to MFH, were used as controls. Twenty-four hours later than the MFH treatment, the cytotoxicity assays were performed. The viability of MDA-MB-231 cells was evaluated after one and two hyperthermia treatments (24 h apart) and normalized to that of the respective controls.

### 2.10. In Vivo Studies

The biodistribution of G-M55 was investigated in four normal nude homozygote female mice, 6–8 weeks old (supplied by Envigo, Bresso, Italy). MRI was performed with a Bruker Biospin 7T scanner (Bruker Biospin, Ettlingen, Germany). Animals were placed in a heated animal bed and inserted in a 7.2 cm internal diameter bird-cage coil under anesthesia (1.5% isofluorane in a mixture of O_2_ and air). G-M55 was injected at a dosage of 4.0 mg Fe/kg (corresponding to 10.3 mg MNPs/kg dissolved in a volume of 150 μL) through the tail vein. Images were acquired before, and 10 min, 30 min, 60 min, 24 h, and 72 h post-injection. The sequences adopted were the following: *T*_2_ RARE sequence with TR = 1200 ms, TE_eff_ = 36 ms, FOV = 35 × 35 mm^2^, MTX = 128 × 128, slice thickness = 1.0 mm.

The efficacy of G-M55 as a MFH agent was investigated in a group of 18 Balb/c *nu/nu* female mice (Harlan Laboratories, Udine, Italy). Mice were maintained under standard environmental conditions (temperature, humidity, and 12 h/12 h light/dark cycle, with water and food ad libitum) and veterinary control, in the University of Verona animal facility. Two million cells were injected subcutaneously into the right flank of each mouse. The size of the tumor was measured every 3 days by MRI, starting from 15 days post-inoculation. After 21 days (when tumor volume reached about 200 mm^3^), the mice were divided into three different intratumoral treatment groups. The first group (CTRL) received saline, the second (G-M55) received G-M55 (1.2 mg Fe/mL, total volume 100 μL), the third group (G-M55 + MFH) received G-M55 (1.2 mg Fe/mL) and MFH treatments. Animals belonging to the third group were exposed to four MFH cycles: immediately, and after 24, 48, and 96 h. Each MFH treatment comprised the application of an AMF for 1200 s, at an intensity of 8.75 kA/m and a frequency of 473.1 kHz.

Tumor size in all mice was measured by MRI before receiving G-M55 (day 0), and 4-, 6-, and 9-days post-injection. Transversal *T*_2_ weighted images were acquired using a RARE 3D sequence with TR = 1200 ms, TE_eff_ = 47.5 ms, NEX = 1, field of view = 25 × 25 × 25 cm^3^, MTX = 256 × 128 × 32, slice thickness = 0.8 mm, FA = 90°, RARE factor = 16. The tumor volume was obtained by manually drawing ROIs slice by slice using the Paravision software. The percentage increase in tumor volume was calculated at each time point as 100 × (V_*t*_ − V_0_)/V_0_, where V_*t*_ is the tumor volume at time *t* and V_0_ is the initial volume.

Individual animal body weight and clinical signs (spontaneous movement, head posture, and social behavior) were monitored. The experimental plan received authorization from the Italian Ministry of Health (protocol number 56DC9.38) and was approved by the local ethical committee. Animal experiments were conducted in full compliance with Italian law (D.L. 4 March 2014 no. 26) and European Union regulations (2010/63/EU).

### 2.11. Histology

Once the in vivo experiments were completed, mice were sacrificed, and relevant tissue (tumor, liver, kidneys, spleen, lungs, stomach, intestine, and heart) was removed for histology. Excised samples were washed with PBS 0.1 M and fixed in Carnoy solution for 2.5 h. Tissues were embedded in paraffin, cut into 8 µm sections with a microtome, and dried at 37 °C for 24 h. Prussian blue staining was performed to evaluate the presence of iron in the tissues: sections were incubated with PB solution (5% hydrochloric acid and 5% potassium ferrocyanide) for 20 min and counterstained with nuclear fast red (Bioptica, Milan, Italy) for 2 min. To evaluate tissue damage and morphology, sections were stained with hematoxylin and eosin. Sections were examined under a light microscope (Olympus BXS1, Hamburg, Germany), equipped with a charge-coupled device camera.

### 2.12. Immunofluorescence

Immunofluorescence was used to observe the overexpression of HSP70 within the cell culture (MDA-MB-231). After two and four MFH treatments, cells were fixed with 4% PFA solution at room temperature, and then with 70% ethanol at −20 °C overnight. After fixation, cells were permeabilized with 0.1% Triton X-100 in PBS for 10 min at room temperature. Subsequently, cells were incubated in blocking solution for 30 min and immunostained with primary Rabbit Anti HSP70 (Abcam 1:50, Cambridge, UK) antibody, overnight at 4 °C. Secondary rabbit antibody (Alexa Fluor^®^ 488) ab150077 was used at 1:100 dilution. Cells were then washed with PBS, counterstained with Trypan Blue (GIBCO 1:10) for 15 s, washed with PBS, stained for 5 min with 1 μg/mL of Hoechst 33342 (Sigma, Burlington, MA, USA) in PBS, and mounted in PBS:glycerol (1:1) for inspection with a LEICA TCS-SP5 inverted confocal microscope. A diode laser was used for Hoechst 33342, with λ = 405 nm; a visible laser for HSP70, with λ = 496 nm; and a He/Ne laser for Trypan Blue, with λ = 543 nm.

### 2.13. Statistical Analysis

Statistical analysis was conducted with MatLab (Mathworks, Natick, MA, USA), then confirmed and plotted using Prism software (9, GraphPad Inc., La Jolla, CA, USA). All data reported in the paper are expressed as mean ± standard error of the mean (mean ± SEM). A two-way ANOVA test was applied to evaluate statistically significant differences with multiple comparisons, using the Tukey-Kramer test. The statistical significance level was placed at *p* < 0.05.

## 3. Results

### 3.1. Structural Characterization and Stability of G-M55

**Figure 1:**
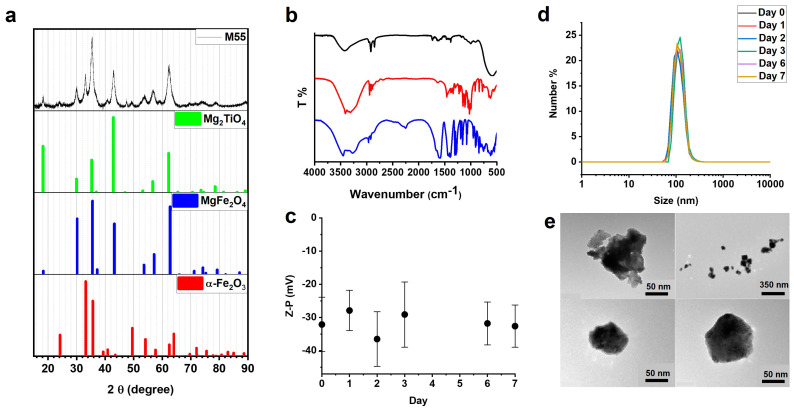
Chemical-physical characterization of M55 and G-M55 nanoparticles. (**a**) Experimental XRPD pattern (black), compared with those of some metal oxides (from ICSD database: Mg_2_TiO_4_ ICSD–65792, MgFe_2_O_4_ ICSD–9939, α-Fe_2_O_3_ ICSD–26410). (**b**) Comparison between FTIR spectra of G-M55 nanoparticles (black line), trisodium citrate (blue line), and glucose (red line). (**c**) Zeta potential of G-M55, monitored for one week. (**d**) Hydrodynamic size distributions of G-M55 throughout the entire week. (**e**) TEM images of G-M55 nanoparticles.

The obtained XRPD experimental pattern is shown in Figure 1a, together with others reported for some iron oxide crystal structures from the ICSD database. A comparison with the literature patterns reveals that the sample under investigation is compatible with a mixture of different iron oxides, such as hematite (α-Fe_2_O_3_), magnesium ferrite (MgFe_2_O_4_), and magnesium titanate (MgTiO_3_). A precise analysis of the different phase contents is not straightforward, due to the nanosized nature of the sample and the strong overlaps of the XRPD reflections of the three above-mentioned crystal phases. Nonetheless, an analysis of the XRPD reflection intensities using a powder diffraction software (SIeve for PDF-4), indicates that a mass ratio of 60:30:10 among the MgFe_2_O_4_, Mg_2_TiO_4_ and α-Fe_2_O_3_ phases, respectively, is reasonably present.

FTIR spectra of the G-M55 NPs, shown in Figure 1b, underline the presence of both citrate ions and glucose molecules, as capping agents, on the surface of the NPs. Figure 1b shows broad bands around 3300 cm^−1^ and 2900 cm^−1^, due to -OH and -CH stretching, respectively, ascribed to glucose and citrate groups. The presence of citrate groups is confirmed by evident features in the 1600–1800 cm^−1^ region (see Figure S1) corresponding to asymmetric stretching of the carboxylate group [12]. The positions of these bands for the NPs show an offset with respect to the free citrate moiety, related to the presence of relevant interactions between the carboxylate groups and the metal ions on the surface. These findings clearly demonstrate the capping of the ligands on the NPs surface. Bands due to other vibrations of the glucose molecular skeleton in the 900–1200 cm^−1^ region (see Figure S1) are observed, consistent with those in the infrared absorbance spectrum of the free glucose molecule (red line in Figure 1b) [12]. Metal–oxygen vibrations, such as Fe-O, Mg-O, and Ti-O ones, are assigned to the strong band around 560 cm^−1^ [13].

Dynamic light scattering (DLS) was used to investigate the colloidal stability of water dispersion of G-M55 through zeta potential and hydrodynamic size measurements (Figure 1c,d). The average zeta potential of −32.1 ± 8.2 mV showed good colloidal stability in aqueous environments. The average hydrodynamic diameter was 113.1 ± 30 nm. Colloidal stability and hydrodynamic size were investigated as a function of time for an entire week. The zeta potential and the size of the nanoparticles remained substantially unvaried, confirming excellent colloidal stability in water, as well as the chemical stability of the coating. In order to further investigate the colloidal properties of the G-M55 NPs in a physiological medium, we considered a dispersion in Dulbecco’s Modified Eagle Medium (DMEM), which is a widely used basal medium for supporting the growth of many different mammalian cells, modified with the addition of 10% of Fetal Bovine Serum and 1% Penicillin-Streptomycin (P/S). A comparison of the hydrodynamic diameter for the G-M55 NPs in water and in a culture medium is shown in Figure S2. Interestingly, the NPs’ average hydrodynamic size notably increases from about 100 nm to 300 nm, indicating a relevant bonding of large molecules as serum proteins, present in the growth medium, to the NPs. The corresponding zeta potential for the NPs dispersion in the growth medium is lower than that measured in pure water (change from −30 mV to −10 mV), confirming the bonding of the large (serum protein) molecules. The increase of the hydrodynamic size is compatible with the formation of a protein corona surrounding the G-M55 NPs, suggesting a possible stabilization in the physiological medium.

TEM images, shown in Figure 1e, are in line with the DLS measurements (Figure 1d), denoting a particle size, as single nanoparticles or as aggregates, from 60 nm to 250 nm.

### 3.2. Magnetic Properties of G-M55

**Figure 2:**
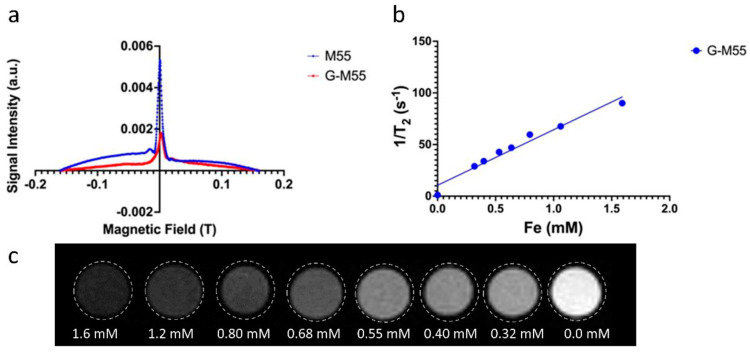
Characterization of G-M55 as a bimodal contrast agent. (**a**) Relaxometry measurement of naked (M55; blue line) and coated (G-M55; red line) nanoparticles, through the MPI RELAX module. This measurement enabled quantification of signal efficiency and of the resolution given by the tracer. (**b**) Plot of transverse relaxation rate (1/T2) vs. Fe concentration in water-dispersed G-M55 phantoms, at 25 °C. The slope of the fitting straight line (53.20 mM^−1^s^−1^) represents the transverse relaxivity coefficient (*r*_2_). (**c**) T2w MR images of phantoms, containing decreasing concentrations of G-M55.

To define the ability of G-M55 to serve as a multimodal contrast agent for both MPI and MRI, two different magnetic characterizations were performed. First, the MPI performance of G-M55 was assessed, in comparison to its naked version (M55) and to a commercially available MPI tracer, VivoTrax (Magnetic Insight Inc., Alameda, CA, USA). Second, the signal efficiency (i.e., the signal per ng of iron), the full-width half-maximum (FWHM), and the approximate resolution were measured through the Magnetic Particle Imager Momentum scanner RELAX module (Magnetic Insight Inc., Alameda, CA, USA). Figure 2a shows the relaxometry measurement of naked (M55) and coated (G-M55) nanoparticles. This measurement allows quantification of signal efficiency and space resolution given by the tracer (see Table 1).

As can be observed from Table 1, even if signal efficiency is approximately 89% (for M55) and 92% (for G-M55) lower than for VivoTrax, resolution, calculated on the basis of the FWHM, is comparable to that of VivoTrax. These observations led us to define M55 and G-M55 as good tracers for MPI. Further developments in the synthesis procedure will be needed to sharpen and intensify the signal efficiency of M55 and its coated version, G-M55.

Figure 2b shows the transverse relaxation rates (1/*T*_2_) of water dispersion containing G-M55 at varying iron concentrations, ranging from 1.6 mM to 0.32 mM. Values of 1/*T*_2_ vs. Fe concentration were fitted, using a straight line whose slope determines the transverse relaxivity of the nanoparticles under investigation (Figure 2b). The transverse relaxivity (*r*_2_) value, 53.20 mM^−1^s^−1^, is comparable to that of a commercial iron oxide MRI contrast agent, such as Endorem^®^, Sinerem^®^, Resovist^®^, Ferumuxytol [14,15,16,17], confirming the potential of G-M55 as a contrast agent for MRI. Figure 2c shows *T*_2_-weighted MR images of G-M55 phantoms. The signal intensity of phantoms decreases with increasing iron concentrations, a characteristic property of a *T*_2_ contrast agent.

**Figure 3:**
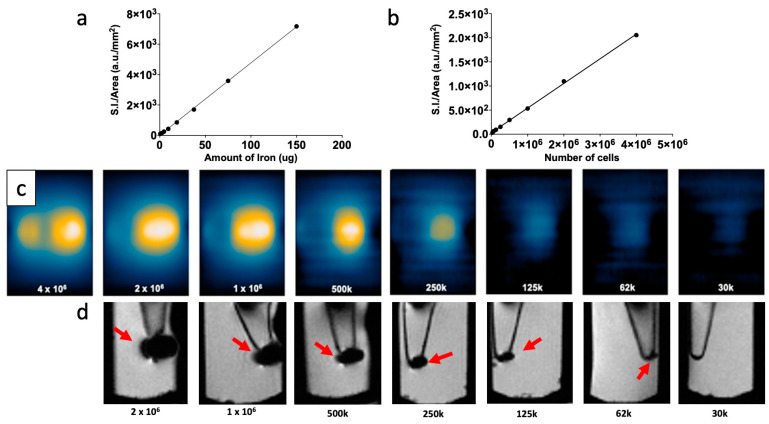
Efficiency of G-M55 as a cellular imaging contrast agent in MPI. (**a**) Signal intensity of G-M55 in water, as a function of the amount of iron per 100 mL of solution. This calibration curve of the tracer defines the ability of M55 to serve as an efficient cell labeling MPI contrast agent. (**b**) MPI signal intensity, as a function of the number of labeled cells. (**c**) MPI images of labeled cells. (**d**) MRI images of labeled cells. Red arrows indicate the hypointense spots attributable to the presence of G-M55 inside the cells.

To evaluate the efficiency of G-M55 as an MPI contrast agent in a biologically significant context, it was used in a cellular imaging experiment testing its capability to label and detect MDA-MB-231 cells by multimodal imaging. The incubation protocol, MPI image collection, and analysis are described above (see Section 2.9). Results are reported in Figure 3. A calibration curve of the tracer was set up by 2D scanning of different MNP concentrations, expressed as iron content (as shown in Figure 3a). This calibration curve was then used to estimate the iron content of cells. Figure 3b shows quantitative values for the MPI signal, obtained in different numbers of cells. The series of images in Figure 3c shows the ability of G-M55 to label cells and make them observable by MPI, up to approximately 30,000 cells. After labeling, cells were also detectable by MRI: images of cell pellets, collected in the bottom of Eppendorf-type tubes are shown in Figure 3d, where the hypointense spots (indicated by arrows) are attributable to the presence of G-M55 inside the cells. Signal voids in MR images are clearly detectable up to 1.25 × 10^5^ cells. Similar tubes containing unlabeled cells were used as controls.

### 3.3. Cytotoxicity, Internalization Mechanism, and In Vitro MFH

**Figure 4:**
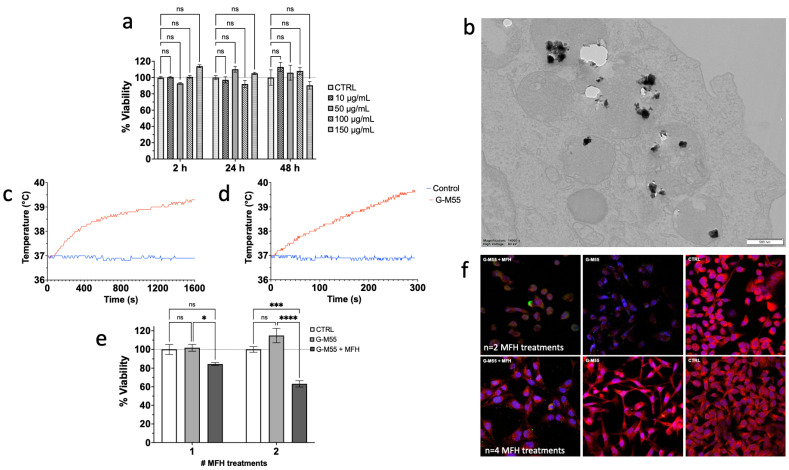
Characterization of G-M55 interaction with MDA-MB-231 cells. (**a**) MTT assay reveals that G-M55 is safe up to a concentration of 150 μg/mL, and up to 48 h of incubation time. The error bar represents SEM over six replicates (ns = nonsignificant). (**b**) Representative TEM image of G-M55 after incubation with cells. (**c**) Thermograms of water-dispersed G-M55 solution, obtained by AMF application using Nanotherics Magnetherm; a plain water solution was used as a control. (**d**) Thermogram of water-dispersed G-M55 solution, upon AMF application by the MPI RELAX module; a plain water solution was used as a control. (**e**) MTT assay of MDA-MB-231 cells after application of one and two treatments (each every 24 h). *: *p* < 0.05, ***: *p* < 0.001; ****: *p* < 0.0001. MFH treatments were performed with the Nanotherics Magnetherm system. The viability of cells was significantly affected by the treatments. (**f**) Upper line: immunofluorescence images of MDA-MB-231 cells after *n* = 2 MFH treatments (G-M55 + MFH) and related controls (G-M55 and CTRL). Lower line: immunofluorescence images of MDA-MB-231 cells, after *n* = 4 MFH treatments (G-M55 + MFH) and related controls (G-M55 and CTRL). Magnification 63×.

Before using nanoparticles as hyperthermia mediators, it is fundamental to evaluate biocompatibility with an in vitro cytotoxicity assay. The toxicity of G-M55 was therefore assessed after 2, 24, and 48 h of incubation with MDA-MB-231 cells, at different concentrations. MTT assay demonstrates that G-M55 is safe up to a concentration of 150 μg/mL for long incubation times (48 h), as shown in Figure 4a. No statistically significant difference was found for the concentrations tested. Considering these results, the highest non-toxic concentration of 150 µg/mL was chosen for MFH in vitro experiments. TEM images obtained in MDA-MB-231 cells demonstrated that G-M55 is internalized mainly via endocytosis and compartmentalized into endosomes (see Figure 4b), consistent with previously reported findings for G-M48 [6].

With a view to a biomedical application as a heat exchange mediator, the SAR of G-M55 was evaluated in two different ways. First, it was assessed by Nanotherics Magnetherm, as described in Gerosa et al. [6]. Second, it was tested by means of the MPI HYPER module. In both measurements, thermograms were collected starting from a specific bulk temperature, about 37.0 °C. This starting condition was chosen to simulate an in vivo experimental setting. Once the good heating ability of the naked nanoparticle (M55) was observed, the measurements were carried out on the coated version, G-M55. The same iron concentration was used to estimate SAR in an aqueous solution, with the two methods. Figure 4c,d shows the thermograms collected in aqueous suspensions of G-M55, using Nanotherics Magnetherm and the MPI HYPER module, respectively. As shown in Figure 4c, a temperature increase of about 4 °C was detected at the concentration used. SAR estimated with Nanotherics Magnetherm was 18 ± 1.0 Wg^−1^, when expressed in terms of Fe mass. In this case, the parameter C = H*f amounted to 4.1 × 10^9^ Am^−1^s^−1^. It is noteworthy that a similar initial slope was determined when thermograms were acquired with the MPI RELAX module, using C = H × f = 4.4 × 10^9^ Am^−1^s^−1^. In the last case, SAR was equal to 20 ± 1.4 Wg^−1^. Considering that similar SAR values were obtained with the two different methods, we have chosen to develop our magnetic hyperthermia strategy using the Nanotherics Magnetherm, because its significantly lower safety criterion, as evaluated by the *C* parameter (see the discussion, below), enables a more reliable and scalable solution for clinical use. In addition, the Nanotherics Magnetherm experimental set-up is readily usable for both in vitro and in vivo treatments.

G-M55 was tested as a hyperthermia mediator on a triple-negative mammary carcinoma cell line, MDA-MB-231, at a concentration of 150 μg/mL. MDA-MB-231 cells were incubated with G-M55 for 24 h and exposed to two cycles of AMF, 24 h apart. Cell viability data after MFH are shown in Figure 4e. Cell viability was significantly affected by AMF application, compared to control conditions (untreated cells and cells treated only with G-M55, but not exposed to MFH). MDA-MB-231 cells treated with G-M55 and exposed to AMF showed a percentage of 84.45 ± 1.67% and 63.15 ± 3.44% viability, after the first and the second MFH treatment, respectively. The percentage of viability for cells treated with G-M55 but not exposed to AMF was about 100% at both time points, thus confirming the safety of nanoparticles at the concentration of 150 μg/mL. The viability of cells subjected to MFH cycles, but not incubated with MNPs, was not different from that of native cells.

To gain insight into the mechanism of cell death, after two and four MFH treatments, cells were processed for an immunofluorescence protocol to evaluate the expression of HSP70, selected as an indicator of cellular stress, including temperature increase. As shown in Figure 4f, the expression of HSP70 is more evident in the samples treated with hyperthermia (G-M55 + MFH) than in controls (G-M55 and CTRL), especially after two MFH treatments. This finding is consistent with an increase in temperature in the samples during MFH treatments, as expected. Surprisingly, the expression of HSP70 in the sample treated with G-M55 and exposed to four MFH treatments is lower than that observed in the sample exposed to two treatments, possibly indicating an effect of thermotolerance induced by multiple AMF exposures.

### 3.4. In Vivo Biomedical Application of G-M55 as a MFH Mediator

**Figure 5:**
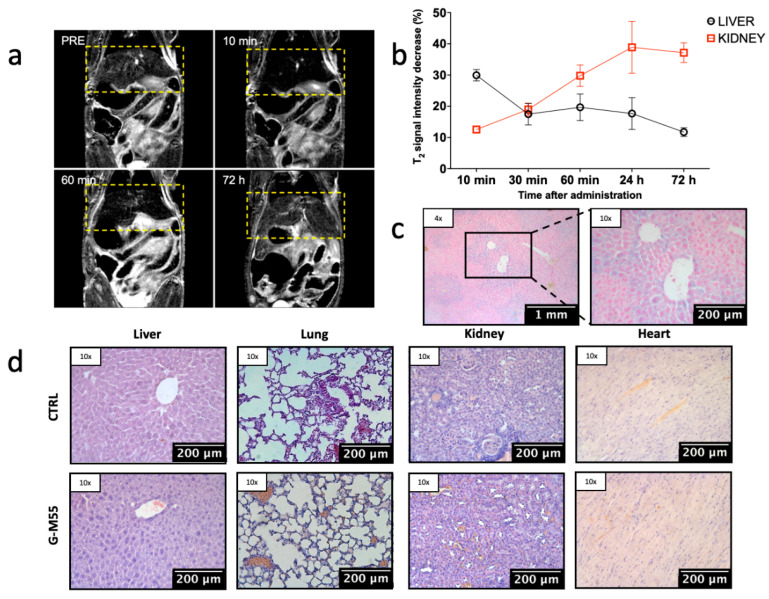
Biodistribution and clearance of G-M55. (**a**) Representative T2w MR images of murine body, acquired basally, and 10 min, 60 min, and 72 h after injection of G-M55. (**b**) Percentage average decrease of SI in liver and kidney at different time points. (**c**) Prussian blue staining of liver tissue, excised 72 h after G-M55 injection. (**d**) Histological slices of main organs, stained with H&E obtained from a representative mouse administered with G-M55 vs. control.

The biodistribution and clearance of G-M55 were investigated in vivo. Figure 5a shows T2w-MR images of a representative mouse body, acquired basally and 10 min, 60 min, and 72 h after injection of G-M55. Signal intensity (SI) in the liver decreased by 29.9 ± 3.7% (Figure 5b) 10 min after injection, and by 11.7 ± 2.8% 72 h after injection, indicating early hepatic accumulation of the nanoparticles followed by clearance. On the other hand, the percentage SI decrease in the kidney went from 12.5 ± 2.3% 10 min after injection, to about 40%, 24 and 72 h after injection, suggesting renal clearance of G-M55. Figure 5c shows histological slices of the liver, excised 72 h after G-M55 injection and stained with Prussian blue. The slight presence of iron in the liver supports the in vivo findings, confirming early NP clearance. To evaluate the safety of G-M55, morphological tissue evaluation of major organs (including liver, lungs, kidneys, and heart) was performed 72 h post-administration (Figure 5d). H&E staining provides evidence of G-M55 biocompatibility in vivo. No alteration in the tissue morphology of relevant organs was detected, as compared to organs of control mice, confirming the absence of systemic toxicity. Specifically, the parenchymal architecture of the liver showed hepatocytes without evidence of steatosis, and was comparable to the control group. Likewise, morphological evaluation of the lungs, kidneys, and heart showed no signs of fibrosis, inflammatory infiltration, or necrosis. Moreover, no significant alteration was detected in individual animal body weight and in clinical signs.

**Figure 6:**
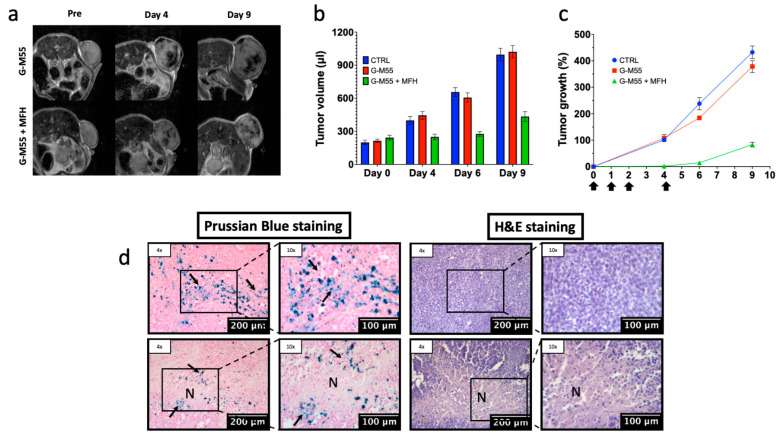
Efficacy of MFH treatment in MDA-MB-231 breast cancer. (**a**) Representative MR images of tumor growth for the group treated with G-M55 (upper line), and the group receiving G-M55 and MFH (lower line), at different time points. The presence of G-M55 is detectable inside the tumor as signal voids. (**b**) Evolution of tumor volume in the CTRL, G-M55, and G-M55 + MFH groups. MFH treatments were performed on days 0, 1, 2, and 4. (**c**) Evolution of tumor growth (%) for the three groups. Black arrows along the time axis indicate times of MFH treatment. (**d**) Prussian blue and H&E staining of tumor tissue. Arrows = Iron. N = necrosis.

The efficacy of intratumoral G-M55 injection in MFH was tested in an experimental breast cancer model. Figure 6a shows MR images of representative mice belonging to the G-M55 and G-M55 + MFH groups, basally and at different time points during the treatment protocol. The presence of G-M55 in the tumor tissue is detectable by MRI as signal voids, consistent with its ability to behave as a negative contrast agent. It is noteworthy that MRI clearly shows the presence of G-M55 within the tumor up to 9 days after injection. In Figure 6b, the tumor volume progression in the three groups is shown on day 0 (prior to G-M55 injection), day 4 (after 4 MFH treatments), day 6, and day 9. The same data are shown in Figure 6c, as percentage tumor growth; here, the timing of MFH treatments is indicated by arrows on the horizontal axis. Starting from day 4, the tumor volume in G-M55 + MFH mice remains significantly lower than in control animals (saline solution and G-M55). In Figure 6d, histological slices of tumor tissue, stained either with Prussian blue (for iron detection) or H&E (for tissue morphology evaluation), are shown for the G-M55 and G-M55 +MFH groups. The blue spots (Figure 6d, arrows) indicate the presence of iron inside the tumor 9 days after G-M55 injection, supporting the in vivo MRI findings (Figure 6a). Absence of evident parenchymal alteration and good preservation of cellular morphology were observed in G-M55 tumor tissue, while G-M55 + MFH tumor tissue shows extensive areas of necrosis (Figure 6d N), attributable to MFH treatment. Differently from other papers from our group [18], in the present experimental set-up, the presence of a closed box with a thermostatic air stream prevented the possibility to acquire IR images.

## 4. Discussion

Thermal therapy, i.e., a local increase of tumor tissue temperature, is widely applied in cancer treatment. Both hyperthermia, i.e., heating to 39–45 °C to induce sensitization to radiotherapy and chemotherapy, and thermal ablation, where temperatures beyond 50 °C destroy tumor cells directly, are frequently applied in clinical practice [19]. Among the different approaches that can be used to induce local temperature increase [3] in tumor tissue, MFH is regarded as a minimally invasive method without systemic toxicity. MFH is based on a limited temperature increase (42–46 °C) of tumor tissue that can lead to apoptosis, avoiding extensive necrosis. It is well known [20], that cancer cells are more sensitive to temperature increase than non-tumoral cells, and that temperatures around 46 °C can usually induce tumor cell apoptosis.

The use of self-regulating temperature materials, which heat only to the T_C_, i.e., the temperature at which a magnetic phase transition from ferromagnetic to paramagnetic occurs, is seen as an undoubted advantage of this technique: it affords an intrinsic safety mechanism, guarding against the risk of damage to surrounding tissues [6,8]. The innovative nanomaterial—developed and patented by MBN, and functionalized by the University of Verona—has a T_C_ that can be modulated by synthesis, providing a temperature-limiting effect during AMF heating. M55, the magnetic core of the NP investigated in the present study, has a T_C_ value of about 95 °C (see Gerosa et al. [6]). Since MFH ideally produces a limited increase in tissue temperature (42–46 °C), this T_C_ value may seem too high. However, recent experimental results have demonstrated that the very marked temperature increase, up to Δ*t* = 45 °C within a distance of 0.5 nm from the MNPs’ surface, falls off exponentially as this distance increases [21]. Therefore, a T_C_ value of about 95 °C could ensure that NPs reach a sufficiently high temperature to be effective MFH agents while remaining intrinsically safe for healthy tissues. Indeed, no damage to tumor-neighboring organs was observed in the present study.

We have demonstrated that G-M55 has diagnostic capabilities in bimodal imaging. G-M55 displays *r*_2_ relaxivity comparable to commercial MRI contrast agents such as Endorem^®^. As far as MPI is concerned, the signal efficiency of G-M55 is about 10% of that of a commercial MPI agent (such as VivoTrax), with comparable spatial resolution. G-M55 thus has good properties as a bimodalcontrast agent. In addition, in a biologically significant context, namely in a cellular imaging experiment, G-M55 allowed the labeling and detection of cells in vitro. Cells labeled by using G-M55 were detectable by both MPI and MRI. MPI allowed the detection of a lower number of cells than MRI, this result being consistent with the greater sensitivity of the former. Although preliminary, this experiment indicates that G-M55 has potential for application in the field of cellular imaging.

Another interesting characteristic of G-M55 is that it can be detected by both MRI and MPI. Moreover, with a view to future clinical applications, the finding that SAR values with standard MFH and MPI measurements are similar is of particular interest for the prospective use of G-M55 as a magnetic fluid hyperthermia agent, in both classical and advanced applications [5]. In addition, the experimental conditions adopted are transferable to clinical protocols.

The timing of the in vivo experiment was decided with potential clinical application in mind—i.e., a single injection of G-M55, followed by multiple applications of AMF in rapid succession. This protocol was very effective in reducing tumor growth rate. Moreover, MRI showed the substantial presence of MNPs within the tumor up to 9 days after injection, enabling delayed AMF without further injections of MNPs.

In this paper, we have examined the application of a synthetic MNP, namely G-M55, in MFH in preclinical studies with reference to both diagnostic and therapeutic capabilities. G-M55 revealed good performances as a bimodal diagnostic contrast agent and as a therapeutic agent in MFH, with either standard or MPI-based systems. Our results demonstrate the efficacy of MFH in the treatment of breast cancer [8,22,23,24].

## 5. Conclusions

M55 belongs to a class of innovative nanomaterials, made up of doped ferrites with the potential for application in tumor therapy, as agents for MFH, and in diagnosis, as contrast agents for MRI and MPI. We have demonstrated the marked efficacy of G-M55 in tumor growth inhibition, by administering a single intratumoral injection followed by repeated AMF treatments in rapid succession. It is noteworthy that SAR values as measured with standard MFH apparatus and MPI methods are similar, opening the way for the application of G-M55 in localized hyperthermia by MPI apparatus. We have demonstrated that G-M55 has diagnostic capabilities in bimodal imaging since it displays r2 relaxivity comparable to commercial MRI contrast agents such as Endorem^®^, as well as good sensitivity/resolution in MPI. In addition, in the biologically significant context of a cellular imaging experiment, G-M55 allowed the labeling and detection of cells in vitro. Overall, our results pave the way for efficient and minimally invasive cancer therapy and diagnosis, by application of a single nanomaterial with self-regulating temperature properties.

## Figures and Tables

**Table 1:** Signal efficiency, FWHM and approximate resolution for M55 and G-M55, compared to VivoTrax.

Nanoparticle	Signal Efficiency (Signal per ng/Fe)	FWHM	Approximate Resolution (mm)
M55 (isopropyl)	1.8	7.5	0.7
G-M55 (water)	1.3	16.4	1.5
Ferucarbotran (VivoTrax)	16.4	11.8	1.1

## Data Availability

The data presented in this study are available on request from the corresponding author.

## Supplementary Materials

The following supporting information can be downloaded at: https://www.mdpi.com/article/10.3390/cancers14205150/s1, Figure S1: Detail of the FT-IR spectrum of G-M55 nanoparticles, sodium citrate and glucose molecules; Figure S2: Hydrodynamic diameter of G-M55 NPs in water (black line) and in DMEM with 10% FBS (red line), using a NPs concentration of 6 mg/mL.
