# Supplementary material for: Doped Ferrite Nanoparticles Exhibiting Self-Regulating Temperature as Magnetic Fluid Hyperthermia Antitumoral Agents, with Diagnostic Capability in Magnetic Resonance Imaging and Magnetic Particle Imaging
Source: Cancers (Basel). 2022 Oct 20;14(20):5150. doi: 10.3390/cancers14205150 (PMC9600860; doi:10.3390/cancers14205150)
Supplement: Supplementary file 1 [file cancers-14-05150-s001.zip › cancers-1906596-supplementary.pdf]

Supplementary Materials

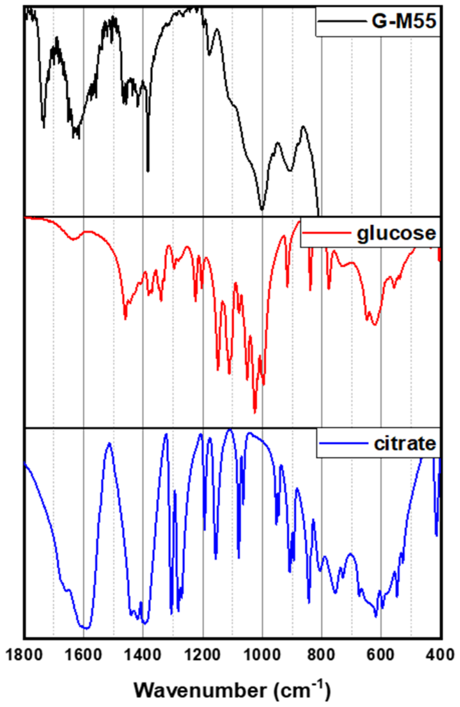


**Figure S1**. Detail of the FT-IR spectrum of G-M55 nanoparticles, sodium citrate and glucose molecules.

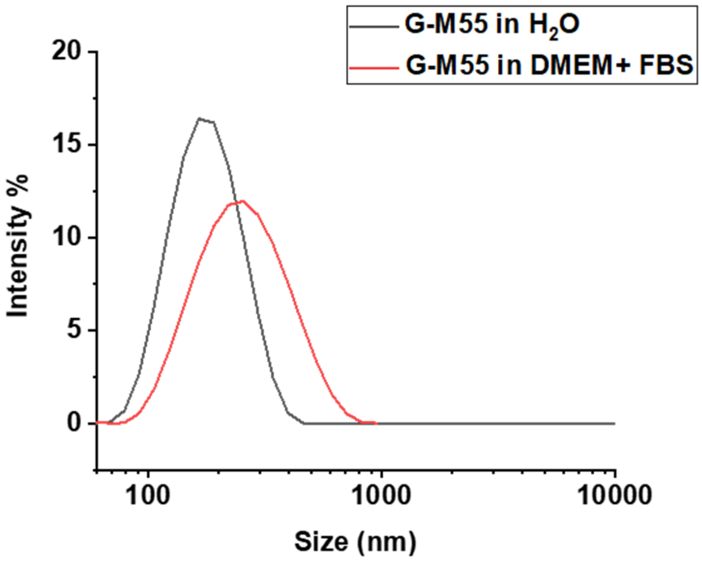


**Figure S2.** Hydrodynamic diameter of G-M55 NPs in water (black line) and in DMEM with 10% FBS (red line), using a NPs concentration of 6mg/mL.
